# Colonic dilation is associated with increased central line associated blood stream infection for patients with intestinal failure

**DOI:** 10.1016/j.intf.2025.100064

**Published:** 2025-06-13

**Authors:** Pamela Emengo, Shweta S. Namjoshi, James C.Y. Dunn

**Affiliations:** a Division of Pediatric Surgery, Department of Surgery, Stanford University, Stanford, CA, USA; b Division of Gastroenterology, Department of Pediatrics, Stanford University, Stanford, CA, USA

**Keywords:** Colon, CLABSI, Intestinal failure, Colonopathy, Enteropathy, Pediatric

## Abstract

**Introduction:**

Management of intestinal failure involves supplemental nutrition and complication minimization to achieve enteral autonomy. Previous research to identify prognostic factors in IF focused on the small bowel, yet little work has been done to associate the colon with IF outcomes. We hypothesize that like small bowel dilation, colonic dilation is associated with intestinal failure morbidity. We also investigate whether a standardized colonic diameter ratio differs from the maximal colonic width in predicting outcomes in children with intestinal failure.

**Material and methods:**

We conducted a retrospective cohort study of all children with gastrointestinal contrast imaging and intestinal failure in our intestinal rehabilitation program between 2013 and 2023. Medical records were reviewed for patient information and imaging review. A colonic diameter ratio was calculated by dividing the maximal colonic diameter by the height of the fifth lumbar vertebra.

**Results:**

Thirty-two patients were assessed based on colonic width and colonic diameter ratio. The median age of patients was 7.0 years (IQR 3 – 11 years). Maximum colonic width correlated positively with age, catheter-associated bloodstream infection, and parenteral nutrition duration. Colonic diameter ratio, on the other hand, did not show a significant correlation with age or parenteral nutrition duration, but had significant positive correlations with catheter-associated bloodstream infection.

**Conclusion:**

Maximum colonic width was significantly associated with increased morbidity in patients with intestinal failure. Increased colonic dilation after normalization by the size of the patient is associated with increased central line associated bloodstream infections.

## Introduction

Intestinal failure (IF) is diagnosed when a patient’s gut function falls below the minimum necessary threshold to absorb nutrients for a period of more than 60 days [1]. Five major pathophysiological conditions give rise to IF: short bowel syndrome (SBS), intestinal fistulas, intestinal dysmotility and pseudo-obstruction, mechanical obstruction, and congenital onset diarrhea and enteropathies (CODEs) [1]. While parenteral nutrition (PN) remains the most important palliative treatment in intestinal failure, prolonged use contributes to the morbidity and mortality of the disease [2].

PN complications include mechanical catheter complications, intestinal failure associated liver disease (IFALD), and impaired quality of life [3]. Catheter-associated bloodstream infections (CLABSI) are a major source of morbidity and mortality for children with intestinal failure and add to the burden of hospitalization and transplantation for those living with IF [4]. In children, CLABSI prevalence has ranged from 2.1 to 9.9 per 1000 catheter days [4].

The ideal management of IF is to support nutrition and growth, minimize complications, and utilize PN to achieve bowel adaptation and enteral autonomy [5]. The probability of weaning a patient with SBS has varied between 50 % for adults and 73 % for children [1]. Hukkinen et al. [6], [7] introduced small bowel dilation as a prognostic factor for children with SBS. While previous research to identify prognostic factors in IF focused on the small bowel and patient characteristics, little work has been done to associate the colon with IF outcomes.

Our previous research showed that colonic length and ileal length are both associated with improved ability to achieve enteral autonomy in patients with SBS [8]. Despite this research, colonic pathology remains understudied in pediatric IF. As such, there is a need to understand if colonic pathology is associated with adverse clinical outcomes in SBS. We hypothesize that like small bowel dilation, colonic dilation is associated with increased morbidity. Like Hukkinen et al. [6], [7] we also investigate whether a standardized colonic diameter ratio differs from the maximal colonic width in predicting outcomes in children with intestinal failure.

## Material and methods

We conducted a retrospective cohort study of all children with IF in our intestinal rehabilitation program between January 2013 and December 2023. Our current work is part of a larger effort to characterize current and past outcomes of patients followed by our institutional Pediatric Intestinal Rehabilitation and Nutrition program. IRB approval for the assessment of long-term outcomes in intestinal failure was obtained and data was stored in our institutional secure electronic Research Electronic Data Capture (REDCap) database.

### Patient selection

**Fig. 1:**
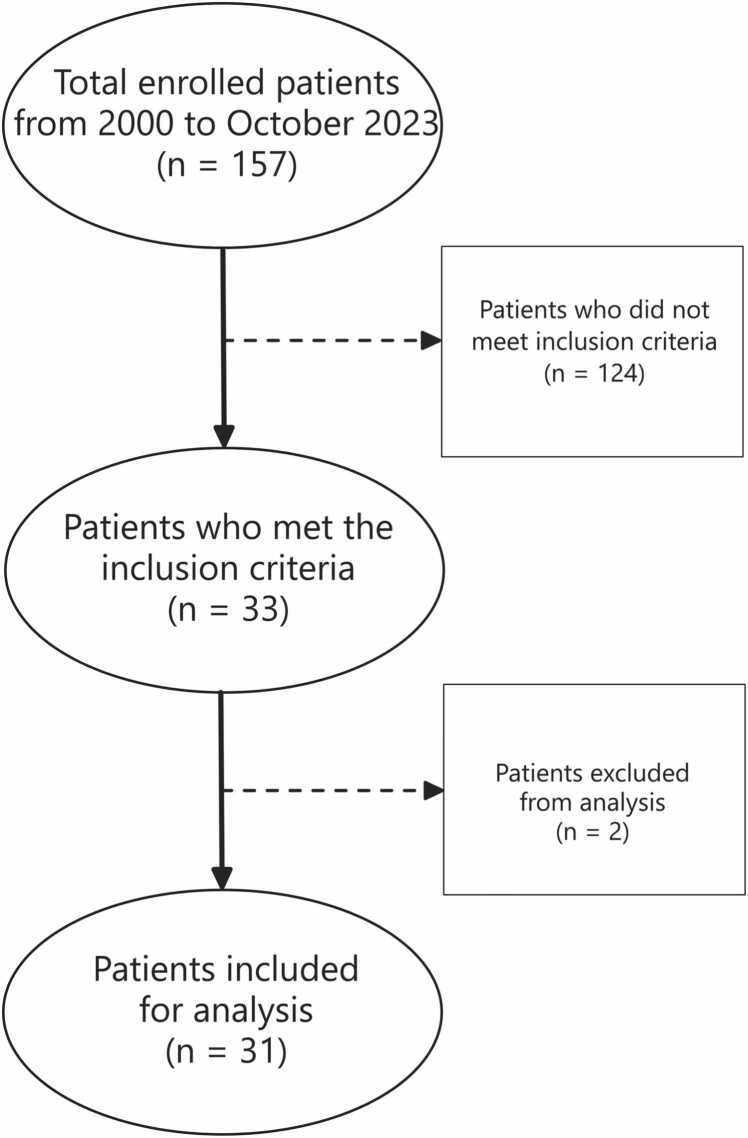
Consort diagram of intestinal failure patients selected for analysis.

Patients were included in the study if they had a diagnosis of IF during the study period and if they had an intestinal contrast study performed that was visible in our electronic medical record (EMR). IF was defined as the loss of functional small bowel length or the dependence of PN for more than 60 days following surgery [9]. Two patients were excluded due to abnormal surgical anatomy (multiple blind limbs of small bowel connected in parallel; history of total colectomy). Patient selection is shown in Fig. 1.

### Variables

Medical records were reviewed and the following variables were collected: patient demographics, etiology of IF, PN dependency, surgical history, underlying anatomy, age at the date of contrast study, post-surgical anatomy, PN duration, and incidence of CLABSI. Patients were further categorized as having achieved enteral autonomy, continuing PN at the time of the study, having undergone intestinal transplantation, deceased, or lost to follow-up. Small bowel lengths were obtained through operative reports, but colonic lengths were not included for analysis due to the significant number of missing measurements in operative reports.

### Parenteral nutrition dependency

The variable PN dependency was obtained as an expert clinician estimate of a percentage of calories from PN compared to the total estimated calories required for age for individual patients. Clinician estimates of PN use at the end of the study period were provided as 0 %, 25 %, 50 %, 75 %, or 100 %.

### Parenteral nutrition duration

The date of PN onset was defined as the first day of documented parenteral nutrition use during the onset of IF. PN duration was calculated by counting the days between the study endpoint and the date of PN onset. For patients who weaned from PN during the time period, a PN free period was calculated and subtracted from the PN duration for each patient. A PN free period was defined as the documented date a patient stopped all PN until either the study endpoint or recurrence of IF.

### Bowel width and bowel diameter ratio

The most recent abdominal contrast imaging was utilized for analysis. Contrast abdominal series were performed using either barium or water-soluble contrast in all patients included in the study. Imaging obtained during the time of an acute intestinal obstruction was not used for the analysis to avoid temporarily dilated bowel. If measurements could not be discerned (n = 14) alternative imaging such as MRI or CT scan was utilized to obtain the measurements. However, contrast imaging was utilized in these patients to confirm anatomy.

Maximal small bowel and colon width perpendicular to the longitudinal axis of the bowel and vertical height of the 5th lumbar vertebra were measured. The width of the largest small and colon were divided by the height of the 5th lumbar vertebra to create the small bowel diameter ratio and colon diameter ratio respectively [10].

### Expected bowel ratio

The percentage expected bowel was obtained by comparing a patient’s most recently measured bowel length (as per operative reports) with normal bowel length for height taken during the hospitalization of the bowel measurement [11].

### Statistical methods

Spearman correlations were used to assess the relationship between colonic dilation and CLABSI, age, and PN duration. A sensitivity analysis was conducted where data was normalized and potential outliers removed (ROUT Q = 1 %) prior to assessing the association between CLABSI and CW and CR. GraphPad Prism Software, version 10.4.0 (San Diego, CA) was used for analysis and figure creation.

## Results

Of the 129 patients in our institutional IF database, 31 met the imaging criteria. Patient characteristics are listed in Table 1 and Table 2. Baseline classifications of intestinal failure were SBS (n = 29), dysmotility (n = 1), or CODEs (n = 1). One patient underwent intestinal transplantation. No patients died during the review period. Our patients predominantly identified as male (64.5 %) and Hispanic/Latino (61.3 %). Overall, our patients predominantly suffered from SBS (93.5 %), had missing ileocecal valves (64.5 %), had a colon resection (54.8 %), did not have a stoma (96.8 %), and did not achieve enteral autonomy (80.6 %). One patient was noted to have received an intestinal transplant (3.2 %).

**Table 1:** Demographics of all patients with intestinal failure.

Variable		Number (N)	Percentage (%)	
Gender				
Female Male		11 20	35.5 64.5	
Race/ Ethnicity				
African American American Indian Asian Caucasian Hispanic Other		3 1 4 3 19 1	9.7 3.2 12.9 9.7 61.3 3.2	
Intestinal Failure Classification				
Congenital Diarrhea and Enteropathy Dysmotility Short Bowel Syndrome		1 1 29	3.2 3.2 93.5	
Presence of ileocecal valve				
Absent Present Not recorded		20 10 1	64.5 32.3 3.2	
Colon resection				
No resection Resection Not recorded		11 17 3	35.5 54.8 9.7	
Intestinal Continuity				
Ileostomy Present	1			3.2
In – Continuity	30			96.8
Enteral Autonomy Achieved				
No Yes		25 6	80.6 19.4	
Other Outcomes				
Intestinal Transplant		1	3.2

**Table 2:** Characteristics of patients with intestinal failure in a retrospective cohort study assessing dilated colonopathy. Baseline patient characteristics. Medians and interquartile ranges (quartile 1, quartile 3). the units for each variable are: age = years, age at imaging study = years, gestational age = weeks, birth weight = grams, remnant small bowel length = centimeters, percent expected small bowel length = percent, small bowel width = cm, colon width = cm, small bowel ratio = unitless, colon ratio = unitless, total CLABSI = episodes, CLABSI per 1000 catheter days = episodes, PN duration = months, PN weaned = percentage.

Patient characteristics		
Variable	Median	IQR (Q1, Q3)
Age	7.0	3, 11
Age at Imaging Study	4.0	1, 10
Gestational Age	33.9	28.6, 36.3
Birth Weight	2150	1470, 2690
Remnant Small Bowel length	37	17.5, 64.5
Percent Expected small bowel length	16.9	5.91, 34.57
Small Bowel Width	2.72	1.82, 4.405
Colon Width	4.09	2.54, 5.11
Small Bowel Ratio	1.54	1.19, 2.72
Colon Ratio	2.64	2.01, 3.30
Total CLABSI	3.0	0, 10.5
CLABSI per 1000 catheter days	1.54	0, 3.69
PN Duration	42	20, 121
PN Weaned	25 %	25 %, 75 %

The median age of patients was 7.0 years, with an interquartile range (IQR) of 3 – 11 years. The median small bowel dilation ratio (SR) of our cohort was 1.54, with an interquartile range of 1.19–2.72. The small bowel dilation ratio (SR) in our cohort was not found to significantly correlate (p > 0.05) with CLABSI, PN duration, maximum colonic width (CW), or the colonic diameter ratio (CR). SR correlated positively with the small bowel width (SW) (r = 0.83, p < 0.05) and negatively with the presence of an ileocecal valve (ICV) (r = - 0.42, p < 0.05).

**Fig. 2:**
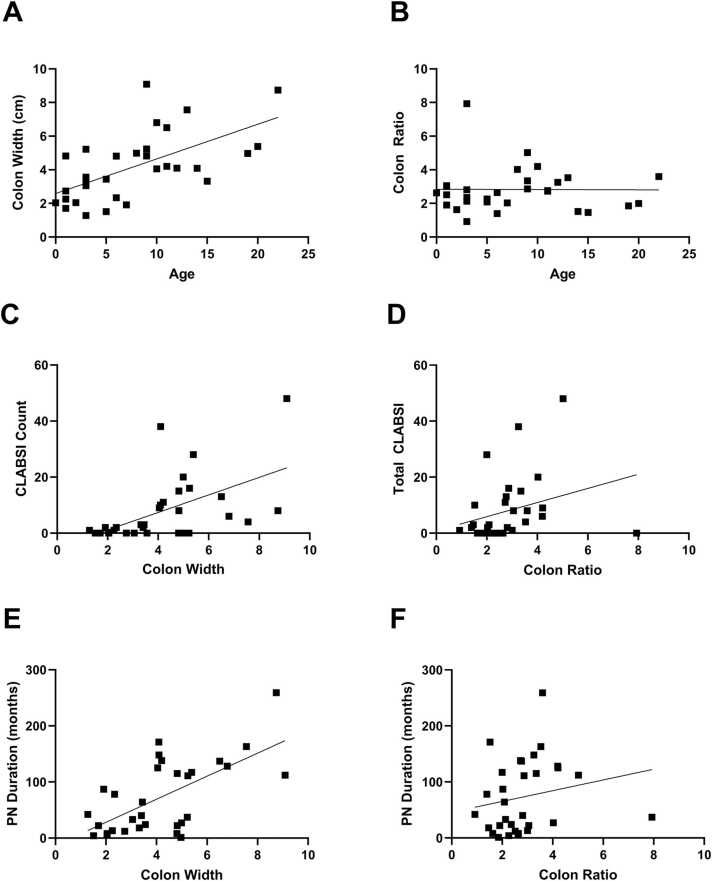
Scatter plots showing the relationship between age, clabsi, pn duration, and colonic dilation in patients with intestinal failure. (A) Shows the maximum colonic width (CW) in centimeters (cm) and age in years. (B) Depicts the colonic diameter ratio (CR) and age in years. (C) Shows the total lifetime CLABSI count and CW in cm. (D) Shows the relationship between the total lifetime CLABSI count and CR. (E) Shows the relationship between the number of months of PN a patient has received and CW in cm. (F) Shows the relationship between the number of months of PN a patient has received and CR.

The median CW was 4.09 cm, with an IQR of 2.54 – 5.11 cm, while the CR was 2.64, with an IQR of 2.01– 3.30. The CW correlated positively with the presence of an ICV (r = 0.37, p < 0.05) age (r = 0.62, p < 0.001), CLABSI (r = 0.61, p < 0.001), and PN duration (r = 0.54, p < 0.001). To normalize the age-related increase in colonic size, we calculated the CR, defined as the maximum colonic width divided by the height of the 5th lumbar vertebra. CR, did not show a significant correlation with the presence of the ICV (r = -0.05, p > 0.05), age (r = 0.10, p > 0.05), or PN duration (r = 0.34, p > 0.05), but had significant positive correlations with total CLABSI (r = 0.41, p < 0.05)(Table 3**,** Table 4**,** Fig. 2). A sensitivity analysis removing potential outliers based on CLABSI was conducted, but did not change the significance of the results for CW and CLABSI (r = 0.56, p < 0.05) or CR and CLABSI (r = 0.40, p < 0.05). Correlations were also performed with those with a colonic resection and found that CW and CR remained correlated with each other (r = 0.65, p < 0.05), but was not associated with CLABSI, PN duration, or age (p < 0.05).

**Table 3:** Spearman correlations between colon diameter, colon ratio, small bowel diameter, expected small bowel length, and ileocecal valve presence. P values are the result of Spearman’s rank correlation pairings. An “*” denotes variables with p < 0.05 values. Percentages May not equal 100 % due to rounding error.

	**CW**	**CR**	**SW**	**SR**	**% SB**	**ICV**
Colon Width (CW)	1.00					
Colon Ratio (CR)	0.64*	1.00				
Small Bowel Width (SW)	0.35	−0.02	1.00			
Small Bowel Ratio (SR)	−0.02	−0.03	0.83*	1.00		
% Small Bowel Remaining (% SB)	−0.12	−0.22	−0.20	−0.12	1.00	
Ileocecal Valve Presence (ICV)	−0.37*	−0.05	−0.60*	−0.42*	−0.06	1.00

**Table 4:** Spearman correlations age, colon diameter, and colon ratio with patients with intestinal failure in a retrospective cohort study assessing dilated colonopathy. P values are the result of Spearman’s rank correlation pairings. An “*” denotes variables with p < 0.05 values.

Spearman Correlations		
Comparison	Spearman Rho	P value
Age vs CW	0.62	0.0002*
Age vs CR	0.10	0.6062
CW vs CLABSI Total	0.61	0.0003*
CR vs CLABSI Total	0.41	0.023*
CW vs PN Duration	0.54	0.0017*
CR vs PN Duration	0.34	0.060

## Discussion

Our study illustrates that colonic dilation, which we term dilated colonopathy, can be associated with IF morbidity, CLABSI, and increased duration of PN dependency. Both CW and CR were associated with the major markers of IF associated morbidity. We believe that we are the first to associate colonic dilation with increased IF morbidity. In addition to previous studies on small bowel dilation, which we term dilated enteropathy for small bowel dilation, dilated colonopathy should be included in our collective understanding of the pathophysiology of chronic IF.

Patients with either an increased CW or an increased CR were correlated with a higher number of CLABSIs. A variety of hypotheses can help explain the association between dilated colonopathy and CLABSI. One hypothesis is that dilated bowel can lead to dysmotility and bacterial stasis, which leads to bacterial overgrowth and translocation [12]. This hypothesis is supported by the reduced morbidity of patients who have received tapering enteroplasty surgeries that minimize the small intestinal diameter [13], [14]. Anecdotally, our 1 transplant patient included in our cohort, received a colonic tapering surgery for his severe colonic dilation. The tapering was performed 1 year prior to intestinal transplantation. In the year preceding tapering, the patient suffered from 6 episodes of CLABSI, but 7 episodes of D-lactic acidosis. One year post colonic tapering, the patient continued to have 6 episodes of CLABSI, but only 1 episode of D-lactic acidosis. While we plan to conduct more research regarding colonic tapering and its potential effects, we hope that our work can promote further investigation of the colon in patients with SBS.

Our findings also suggest that dilated colonopathy may be associated with impaired weaning of PN and the presence of an ICV. Although PN duration and ICV were significantly associated with CW, both were nonsignificant with CR. While ICV and PN duration approach significance with CR, a small sample possibly precluded results.

High CW was predictably associated with age in our study. This has been demonstrated by the literature that shows bowel caliber increases as one ages [15]. However, age was not associated with the CR which suggests that the use of the L5 vertebral height was effective in normalizing age-related colonic growth. The use of the L5 vertebrae has been described in previous works to standardize small bowel measurements [6], [7], [10], but this is the first work to show effectiveness with colonic measurements. Future studies should assess how the L5 vertebral height correlates with validated anthropometric measures like height, body surface area, or developmental percentiles as a proxy for growth scaling. [16].

The SR of our cohort did not correlate with CW, CR, CLABSI, or PN duration. The lack of correlation between SR and our variables is predictable and consistent with previous literature showing that outcomes were unfavorable when the SR exceeds 2.0 [6], [7]. Given that our median ratio was 1.54, our patients fall under the threshold where we would see the effects of small bowel dilation. Moreover, since previous risk factors for worsened IF morbidity were not found to be a significant factor in our cohort, this strengthens our belief that dilated colonopathy played a central role in CLABSI formation.

The overall survival of our cohort (100 %) was above what was quoted in the literature and found in an international intestinal failure registry (IIFR) [17], [18]. Intestinal failure survival rates have been reported as 93 % after 1 year, 71 % after 5 years, 59 % after 10 years, and 28 % after 20 years [18]. Two notable differences in our cohort are age and enteral autonomy achievement. Outcome literature in SBS or IF generally focuses on outcomes in children younger than those in our cohort. For example, in a similar work looking at SB dilation, all patients were less than 2.7 years [6]. Regarding enteral autonomy, compared to the patients in the IIFR, who had a 2-year follow-up 52 % had achieved autonomy, while in our dilated colon cohort only 22 % achieved enteral autonomy [17]. However, if we consider patients with a CW higher than the average of our cohort (4.19 cm) as dilated, we see interesting differences. 13.3 % of patients with dilated colonopathy were weaned from PN, while 25 % were able to be weaned without dilated colon. In the context of our patients in the ongoing prospective arm of the IIFR, when compared to our cohort of children without dilation who are currently included in the ongoing prospective arm of the IIFR, the weaning rates for those with dilated colonopathy are far lower than that of children in our current IIFR cohort; children at our institution, in the IIFR cohort, currently have a 3-year enteral autonomy rate of 100 % and a 1-year enteral autonomy rate of 35 %.

These differences represent a selection bias in children with IF in general and in our study; younger children who wean from PN do not present to care for imaging nor symptoms; children who require imaging for gastrointestinal symptoms tend to be older and on PN. Surgical patients or more morbid patients with more severe IF complications are more likely in our institution to receive contrast imaging to calculate a small bowel dilation ratio or for SBS surgical planning. By excluding patients without imaging, we have excluded younger patients, less morbid patients with IF, and excluded those who gained enteral autonomy at a faster rate.

Despite these differences and potential selection bias, the characteristics of our patients remain generalizable to those described in the literature. Most of our patients suffered from SBS as their underlying etiology of IF. This is consistent with the literature that describes SBS as the most common cause of IF in children [2], [5], [17], [18], [19]. The IIFR data also reported a median birth weight of 2.1 kg, a median small bowel length of 40 cm, a 21 % expected small bowel length for age, 41 % ileocecal valve resection, and CLABSI rates of 1.9 per 1000 catheter days [17]. Our patients had a median birth weight of 2.1 kg, a median short bowel length of 37 cm, a median estimated percent small bowel of 16.9 %, and a CLABSI rate of 1.54 per 1000 catheter days. Overall, we can see that our patients had slightly fewer rates of CLABSI. This suggests that our cohort has partial generalizability and has similar characteristics to those in other large, international cohort studies of children with IF.

In our institution, few practitioners consider diagnosing colonic abnormalities or recording colonic data in patients with IF. Although not highlighted in the study, only 5/31 (16 %) of patients in the study had operative reports that detailed the length of the colon. In comparison 24 of 31 (77 %) recorded small bowel length measurements. While other papers have highlighted the importance of the colon in IF or offered methods to calculate the expected colonic ratio, colonic analysis was limited by our source data [8], [11]. Another limitation is that in our institution, contrast studies are often optimized to visualize the small bowel or upper gastrointestinal tract. Poor timing of contrast injection likely leads to an under-distension of the diseased bowel, and contrast enemas often obscure the vertebral measurements. When considering these limitations, our results may underestimate associations between colonic dilation and IF outcomes. Thus suggesting, that the true bowel dilation ratios or measurements are likely to be larger than stated in this paper.

Our findings were limited by the retrospective nature of our analysis, which makes it challenging to establish temporality or causality with outcomes such as PN weaning or CLABSI incidence. Although part of a larger prospective cohort analysis, imaging was only obtained for standard of care, not exclusively for research purposes. Moreover, patients did not have contrast imaging pre-, during-, and post-dilation. In addition, we were limited by the small sample size of our patients. For certain subcategories of patients such as the discontinuity vs in-continuity, we only had 1 patient in discontinuity. At our institution stoma reversals are performed as soon as safely possible, which limits our ability to generalize our findings. Also due to the limited sample, we were unable to perform regression analysis to further account for potential confounding variables. However, even with potential outliers removed, the relationship between CLABSI and dilated colonopathy remained significant. As this is one of the first of its kind of studies, this work can lay the foundation for future studies further assessing colonic dilation.

## Conclusion

The importance of the colon is understated in IF literature. By analyzing colonic dilation as a risk factor for IF complications, we will be able to better characterize the pathophysiology of IF and develop innovative medical and surgical interventions. Our study, to our knowledge, is the first to illustrate that dilated colonopathy can be associated with IF morbidity, increased CLABSI, and increased duration of enteral nutrition. Future studies should replicate these findings in a prospective sample of infants to see how colonic dilation develops in infants over time, with respect to the clinical characteristics obtained, and assess the utility of colonic tapering in treating the complications outlined in this study.

## CRediT authorship contribution statement

**Pamela Emengo:** Writing – review & editing, Writing – original draft, Visualization, Software, Project administration, Methodology, Investigation, Formal analysis, Data curation, Conceptualization. **Shweta S. Namjoshi:** Writing – review & editing, Supervision, Resources, Project administration, Methodology, Data curation, Conceptualization. **James C.Y. Dunn:** Writing – review & editing, Supervision, Project administration, Methodology, Data curation, Conceptualization.

## Patient’s / Guardians’ consent

Not applicable.

## Ethical statement

IRB approved, protocol number 48468.

## Declaration of Generative AI and AI-assisted technologies in the writing process

Not used.

## Funding

This research did not receive any specific grant from funding agencies in the public, commercial or not-for-profit sectors.

## Declaration of Competing Interest

The authors declare that they have no known competing financial interests or personal relationships that could have appeared to influence the work reported in this paper.
