# Supplementary figures and images for: Colonic dilation is associated with increased central line associated blood stream infection for patients with intestinal failure
Source: Intest Fail. 2025 Jun 13;5:100064. doi: 10.1016/j.intf.2025.100064 (PMC12851260; doi:10.1016/j.intf.2025.100064)

**A**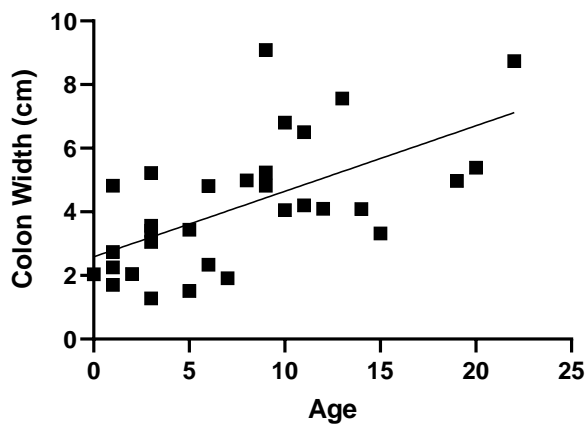**B**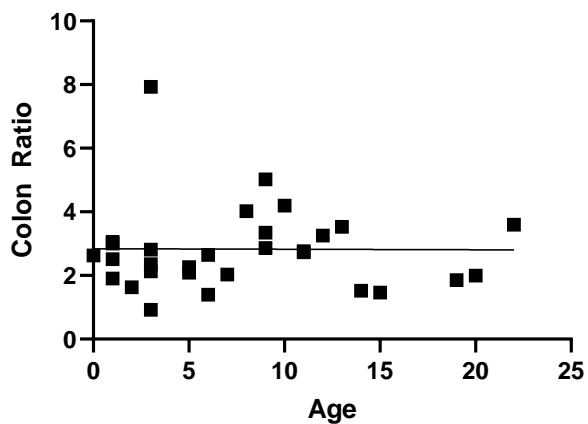**C**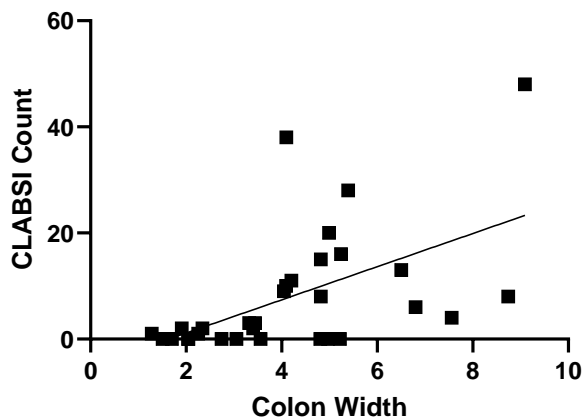**D**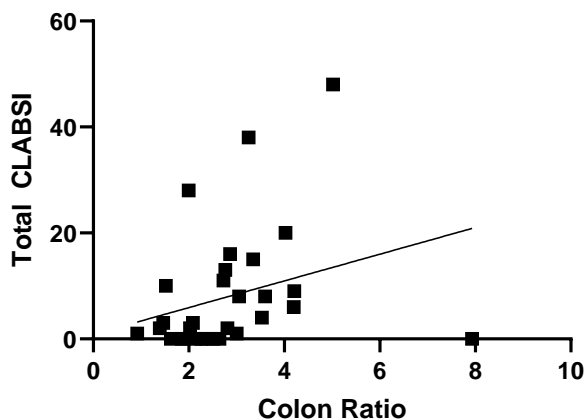**E**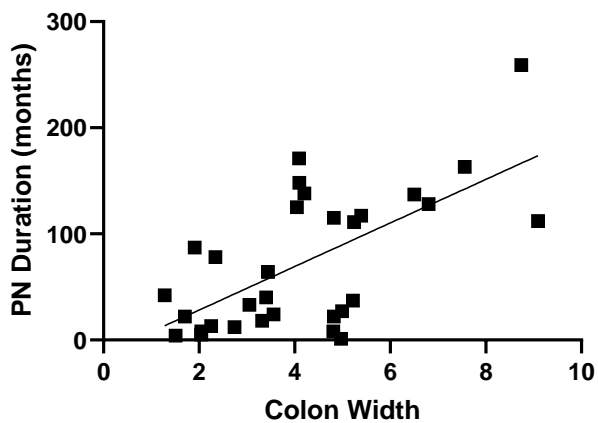**F**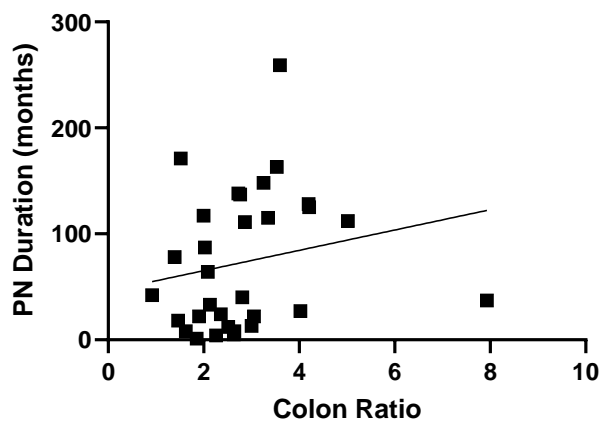

Supplement: Supplementary file 1 — Supplementary material [file mmc1.pdf]

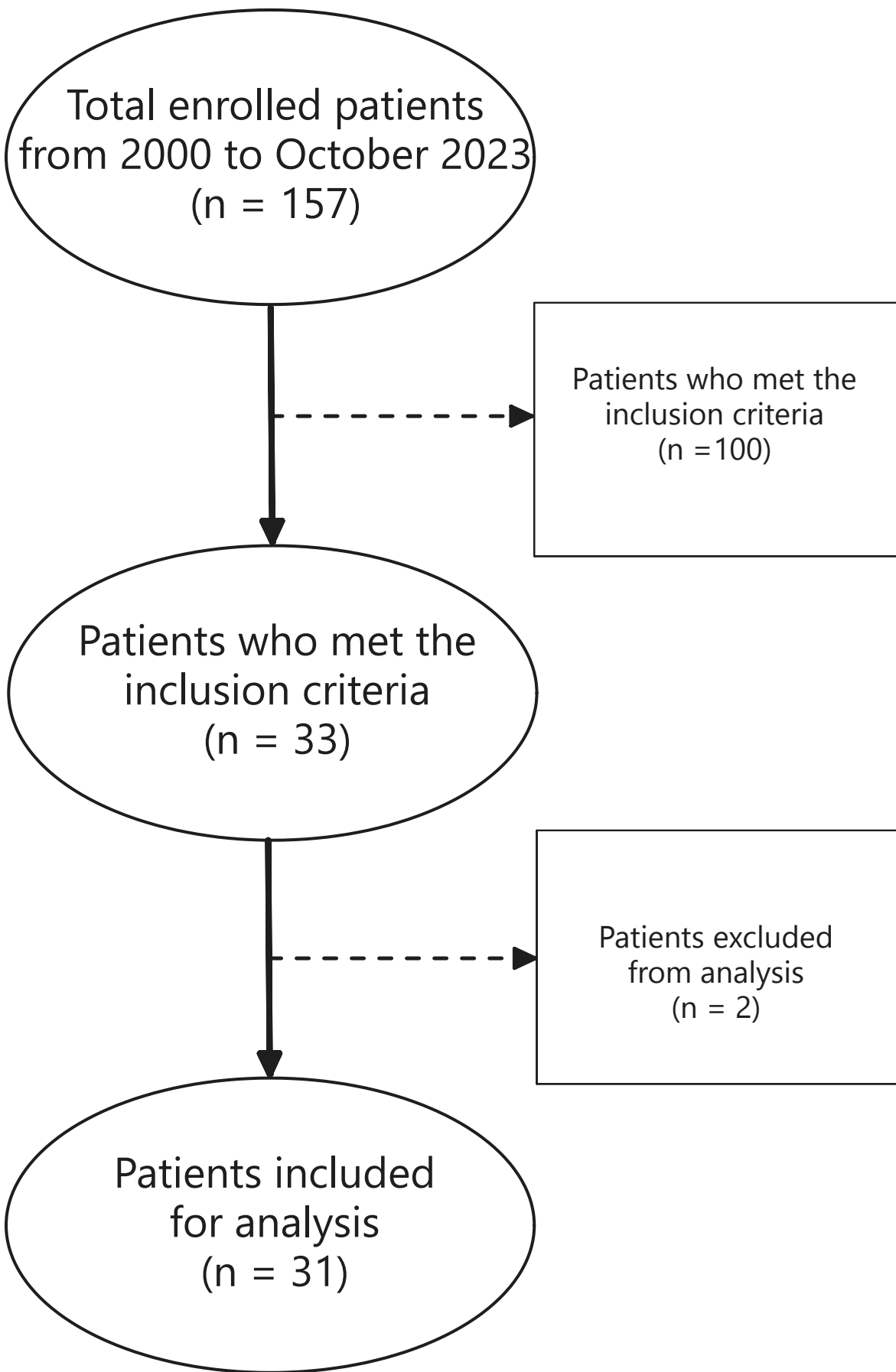

Figure 2. Flow diagram of patient selection

Supplement: Supplementary file 2 — Supplementary material [file mmc2.pdf]
